# Flow cytometry-based quantification of targeted knock-in events in human cell lines using a GPI-anchor biosynthesis gene *PIGP*

**DOI:** 10.1042/BSR20212231

**Published:** 2021-12-08

**Authors:** Md. Lutfur Rahman, Toshinori Hyodo, Muhammad Nazmul Hasan, Yuko Mihara, Sivasundaram Karnan, Akinobu Ota, Shinobu Tsuzuki, Yoshitaka Hosokawa, Hiroyuki Konishi

**Affiliations:** Department of Biochemistry, Aichi Medical University School of Medicine, Nagakute, Aichi 480-1195, Japan

**Keywords:** CRISPR/Cas, genome editing, genome engineering, GPI anchor, PIGP, targeted knock-in

## Abstract

Targeted knock-in supported by the CRISPR/Cas systems enables the insertion, deletion, and substitution of genome sequences exactly as designed. Although this technology is considered to have wide range of applications in life sciences, one of its prerequisites for practical use is to improve the efficiency, precision, and specificity achieved. To improve the efficiency of targeted knock-in, there first needs to be a reporter system that permits simple and accurate monitoring of targeted knock-in events. In the present study, we created such a system using the *PIGP* gene, an autosomal gene essential for GPI-anchor biosynthesis, as a reporter gene. We first deleted a *PIGP* allele using Cas9 nucleases and then incorporated a truncating mutation into the other *PIGP* allele in two near-diploid human cell lines. The resulting cell clones were used to monitor the correction of the *PIGP* mutations by detecting GPI anchors distributed over the cell membrane via flow cytometry. We confirmed the utility of these reporter clones by performing targeted knock-in in these clones via a Cas9 nickase-based strategy known as tandem paired nicking, as well as a common process using Cas9 nucleases, and evaluating the efficiencies of the achieved targeted knock-in. We also leveraged these reporter clones to test a modified procedure for tandem paired nicking and demonstrated a slight increase in the efficiency of targeted knock-in by the new procedure. These data provide evidence for the utility of our *PIGP*-based assay system to quantify the efficiency of targeted knock-in and thereby help improve the technology of targeted knock-in.

## Introduction

Targeted knock-in is a methodology of genome engineering with which a specified DNA element or a base substitution is introduced into a predetermined position of the genome. Because targeted knock-in allows for modifications of the genome exactly as designed, this methodology is considered promising for future applications in various fields of life sciences, including clinical medicine and agriculture [1–4]. Recently, the efficiency of targeted knock-in was dramatically improved by the application of genome editing technologies such as the CRISPR/Cas systems [5] to enhance homology-directed DNA recombination [6,7]. However, efforts are still being made to improve the efficiency, precision, and specificity of CRISPR/Cas-assisted targeted knock-in [8–12].

To modify the procedure of targeted knock-in and improve the efficiency achieved, we first need a molecular system that allows for the sensitive and quantitative detection of targeted knock-in events. We previously developed such a molecular system in which an inactive mutant *PIGA* gene in a human cell line is corrected to a wild-type gene by targeted knock-in [13]. The *PIGA* gene, located on the X chromosome, is essential to biosynthesize glycosylphosphatidylinositol (GPI) anchors which, after being synthesized, are distributed over the cell membrane [14,15]. Thus, cells carrying wild-type but not mutant *PIGA* are sensitive to aerolysin, a cytolytic toxin that binds to GPI-anchored proteins and forms pores on cell membranes. *PIGA*-disrupted cells are therefore selectable with a precursor of aerolysin, namely proaerolysin [16,17]. In addition, cells carrying inactive and reverted *PIGA* can be counted by flow cytometry (FCM) detecting fluorescently stained GPI anchors on the cell surface [18,19]. This latter assay, named *PIGA* correction assay, allowed us to sensitively measure the efficiency of targeted knock-in occurring within an endogenous human gene with a simple, high-throughput, and relatively inexpensive procedure [13].

Despite the advantages of the FCM-based quantification of targeted knock-in events elicited within an endogenous gene, to the best of our knowledge, the *PIGA* correction assay has been the only established monitoring system achieving all the above-mentioned benefits. To eliminate possible gene locus-specific effects and experimental artifacts unique to the *PIGA* correction assay, a second system measuring the efficiency of targeted knock-in based on a different endogenous platform gene is critical. Accordingly, in the present study, we developed a distinct FCM-based molecular system that allows us to quantitatively monitor the correction of an inactivating mutation in two near-diploid human cell lines, using *PIGP* as a platform gene. *PIGP* is another GPI-anchor biosynthesis gene whose product forms a complex with PIGA [20]. We also employed the resultant *PIGP*-based assay (hereafter referred to as *PIGP* correction assay) in an attempt to improve the procedure of tandem paired nicking (TPN) [13], a recently developed nick-based strategy for precision targeted knock-in.

## Materials and methods

### General molecular biology techniques

Extraction of genomic DNA (gDNA) was conducted using the PureLink Genomic DNA Mini Kit (Thermo Fisher Scientific, Waltham, MA, U.S.A.). Polymerase chain reaction (PCR) solution was prepared using KAPA HiFi HotStart ReadyMix (Roche, Basel, Switzerland) as per the manufacturer’s protocol, and amplification was performed in a Veriti thermal cycler (Thermo Fisher Scientific). AE-6932GXES Printgraph (ATTO, Tokyo, Japan) was used for the digital imaging of DNA fragments fractionated on agarose gels. Sequencing samples were prepared using the BigDye Terminator v3.1 Cycle Sequencing Kit (Thermo Fisher Scientific) and a Veriti thermal cycler (Thermo Fisher Scientific) and analyzed by a 3500 genetic analyzer (Thermo Fisher Scientific).

### Plasmids

The plasmids pSpCas9(BB)-2A-GFP (PX458) (#48138), pSpCas9(BB)-2A-Puro (PX459) V2.0 (#62988), and pSpCas9n(BB)-2A-Puro (PX462) V2.0 (#62987) were provided by Dr Feng Zhang (Broad Institute) via Addgene [21]. PX459 and PX462 were used to create plasmids expressing Cas9 nucleases and Cas9 nickases, respectively, targeted to genomic sequences shown in Supplementary Table S1. PX458 was transfected into cells, as is, to determine transfection efficiencies.

To create dual single guide RNA (sgRNA) vectors, sgRNA expression cassettes on PX462-based vectors (strings of a U6 promoter, an inserted spacer sequence, and a sgRNA scaffold with a transcription stop signal) were PCR-amplified using a pair of primers shown in Supplementary Table S2. The resultant PCR products were digested with XbaI and inserted into the XbaI site of different PX462-based vectors in such a way that two sgRNA expression cassettes are placed in the same orientation.

Donor plasmids bearing wild-type *PIGP* homologous regions (Donor-*PIGP*ex3 and Donor-*PIGP*ex2) were constructed by PCR amplifications of the *PIGP* genomic region and the insertion of the PCR products into pBluescript II KS(+) (Agilent Technologies, Santa Clara, CA, U.S.A.), using restriction enzymes whose recognition sites were situated within PCR primers (Supplementary Table S2). The *PIGP* mutations shown in Supplementary Figures S1 and 2 were incorporated into the above-described wild-type donor plasmids (Donor-*PIGP*ex3 and Donor-*PIGP*ex2, respectively) to create donor plasmids bearing mutant *PIGP* homologous regions.

### Cell culture and FCM-based experiments

The DLD-1 and HCT116 cell lines were obtained from American Type Culture Collection (ATCC nos. CCL-221 and CCL-247, respectively). DLD-1 and its derivative clones were cultured in Roswell Park Memorial Institute 1640 medium (Fujifilm, Tokyo, Japan) supplemented with 5% fetal bovine serum (FBS; Merck, Darmstadt, Germany) and 1% penicillin and streptomycin (P&S; Fujifilm). HCT116 and its derivative clones were cultured in McCoy’s 5A (modified) medium (Thermo Fisher Scientific) supplemented with 5% FBS and 1% P&S. Cell lines were maintained at 37°C with 5% CO_2_ in a humidified MCO-175 incubator (PHCbi, Tokyo, Japan). The plasmids were transfected into DLD-1 and HCT116 cells by electroporation using a 4D-Nucleofector System (Lonza, Basel, Switzerland) according to the manufacturer’s instructions. Selection with puromycin (Merck) was conducted for 48 h at concentrations of 3.0 μg/ml (DLD-1) and 0.4 μg/ml (HCT116).

To conduct FCM analyses and cell sorting in the present study, cells were detached with Accutase (Innovative Cell Technologies, San Diego, CA, U.S.A.) and stained with fluorescent-labeled inactive toxin aerolysin (FLAER [18,19]; Cedarlane, Ontario, Canada) at a concentration of 0.1 µg/ml dissolved in phosphate-buffered saline containing 0.25 mM EDTA and 0.4% FBS. Cells were then analyzed using an LSRFortessa X-20 Flow Cytometer (BD Biosciences, Franklin Lakes, NJ, U.S.A.) based on FL1-A (530 ± 15 nm)/FL2-A (586 ± 7.5 nm) dot plots under 488-nm/561-nm laser excitation. Cell sorting was performed using FACSAria III (BD Biosciences).

### Creation of *PIGP* reporter clones

Supplementary Table S1 indicates the sequences of Cas9 nuclease target sites employed to create the initial heterozygous *PIGP* deletion in both cell lines (*PIGP*-nuclease-A and *PIGP*-nuclease-B), as well as those used to extend the deletion in HCT116 (*TTC3*-nuclease-C and Junction-nuclease-D).

Analytical PCRs probing the initial heterozygous *PIGP* deletion were performed with the following primer pairs: *PIGP*int2-F1 and *PIGP*int2-R2, *PIGP*downstream-F1 and *PIGP*downstream-R1, and *PIGP*int2-F1 and *PIGP*downstream-R1. Analytical PCRs probing the creation of the extended heterozygous *PIGP* deletion in HCT116 were performed with the following primer pairs: *TTC3*int1-F and *TTC3*int1-R, *PIGP*int2-F1 and *PIGP*int2-R2, *PIGP*downstream-F1 and *PIGP*downstream-R1, and *TTC3*int1-R and downstream-R1. Two reverse primers were used to amplify the joined deletion ends because *PIGP* and *TTC3* are situated in head-to-head configuration. The primer sequences are shown in Supplementary Table S2.

**Figure 1:**
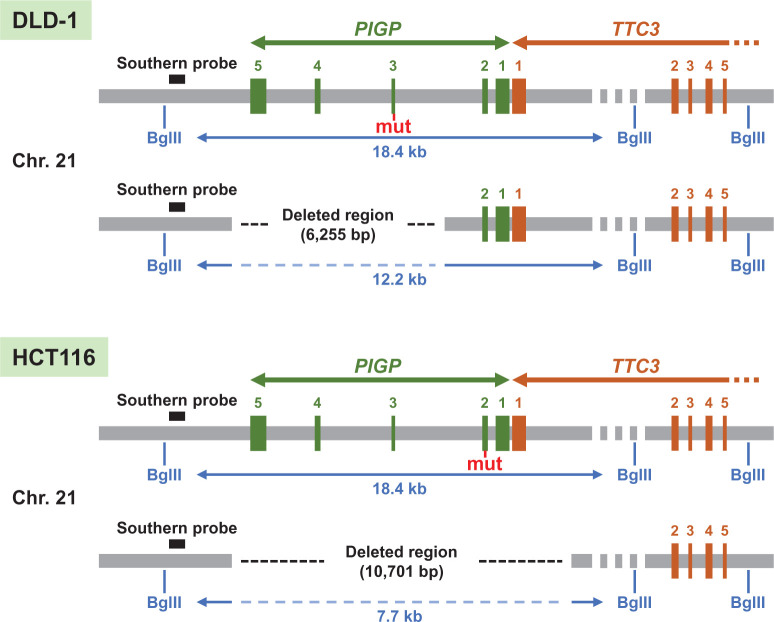
Schematic maps of engineered gene loci in *PIGP* reporter clones Genomic regions surrounding heterozygous deletions introduced in DLD-1-derived (top) and HCT116-derived (bottom) reporter clones are depicted. Numbers in green and brown indicate *PIGP* and *TTC3* exons, respectively. Blue bars and letters indicate the positions of BglII recognition sites and distances between these recognition sites. mut: truncating mutation.

Southern blot analysis to confirm the heterozygous deletions was performed as previously described [13,22,23], except that the BglII restriction enzyme was used to digest gDNA. A probe was produced using a primer pair amplifying the region downstream from the last *PIGP* exon (Figure 1 and Supplementary Table S2).

**Figure 2:**
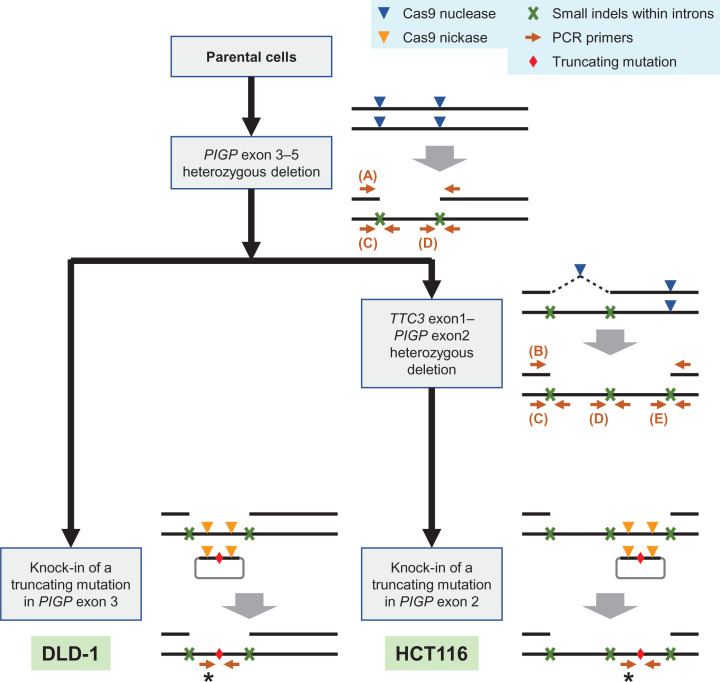
A flow chart depicting the construction of reporter clones The flow chart is shown together with explanatory schemes for respective cell engineering steps. Heterozygous deletions were created exploiting the fact that two adjacent DSBs introduced by Cas9 nucleases on the same chromosome sometimes elicit a genomic deletion between DSBs and sometimes create two indels at the cleaved sites. In HCT116, a 10.7 kb-long heterozygous deletion was introduced by 2-round sequential deletions of 6.3- and 4.4-kb neighboring regions. Truncating mutations were incorporated into the genome by the TPN strategy using Cas9 nickases. Panels (A–E) noted in the schemes represent analytical PCRs whose results are shown in Figure 3A–E. Asterisks indicate PCR amplifications encompassing the knock-in sites, and the nucleotide sequences amplified by these PCRs are shown in Figure 4B.

To create the *PIGP* reporter clones, an inactivating mutation was introduced into the remaining *PIGP* allele in the *PIGP*-heterozygous clones by TPN-based targeted knock-in using the following Cas9 nickases: *PIGP*-nickase-1 and 3 for the DLD-1-derived clone, and *PIGP*-nickase-6 and 7 for the HCT116-derived clone (Figure 2 and Supplementary Table S1).

### *PIGP* correction assay

The *PIGP* correction assay (reversion of mutant *PIGP* allele to a wild-type allele) was initiated by transfecting *PIGP* reporter clones (1 × 10^5^ cells) with a donor plasmid bearing a wild-type *PIGP* sequence along with a Cas9 nuclease or tandem paired Cas9 nickases shown in Supplementary Table S1. Donor-*PIGP*ex3 and Donor-*PIGP*ex2 were employed as donor plasmids for transfecting DLD-1-derived and HCT116-derived *PIGP* reporter clones, respectively. Transfected cells were incubated for three days to allow the production of functional *PIGP* protein from the corrected *PIGP* allele and subsequent GPI-anchor synthesis. Cells were then stained with FLAER and analyzed by FCM to determine the percentage of FLAER-positive cells.

To obtain FLAER-positive ratios, the percentages of FLAER-positive cells in the respective samples were divided by the transfection efficiency determined by transfecting the *PIGP* reporter clones with PX458.

### Statistical analysis

EZR software [24] version 1.54 was used for the statistical analyses performed in the present study. The efficiencies of targeted knock-in achieved using dual sgRNA vectors and those using PX462-based expression vectors were compared in DLD-1-derived and HCT116-derived *PIGP* reporter clones, separately. Statistical analyses were conducted applying two-way repeated measures analysis of variance using ‘vector’ (1 µg each of PX462-based expression vectors, 1 µg of dual sgRNA vector, and 2 µg of dual sgRNA vector) and ‘sgRNA pair’ (two pairs) as two independent variables. After no interactions between these independent variables were found, a Bonferroni *post hoc* test was performed to detect significant differences among three ‘vector’ levels.

## Results

### Creation of a DLD-1-derived reporter clone with which to monitor the correction of a *PIGP* mutation

GPI-anchor biosynthesis is governed by a molecular system comprised of approximately 20 proteins [25]. Many of these proteins are essential for the synthesis of GPI anchors and could therefore be exploited as platforms to monitor the efficiency of gene editing using a cellular phenotype as a surrogate marker, i.e*.* abrogation of one of these genes would result in the loss of GPI anchors from the cell surface. However, among the genes known to be required for GPI-anchor biosynthesis, *PIGA* is the only gene located on the X chromosome [15]. Therefore, to establish a reporter system to monitor gene editing events using a GPI-anchor biosynthesis gene other than *PIGA* as a platform, we planned to delete an allele of a GPI-anchor biosynthesis gene on an autosome so that the absence of GPI anchors on the cell surface determined by FCM analyses can correctly report the frequency of edits created on the other allele. We chose *PIGP* (chromosome 21), the product of which forms a complex with PIGA and plays an essential role in GPI-anchor biosynthesis [20], as the substrate of our genetic manipulation (Figure 1). DLD-1, a near-diploid human cell line, was employed as the first platform cell line for the genetic manipulation.

**Figure 3:**
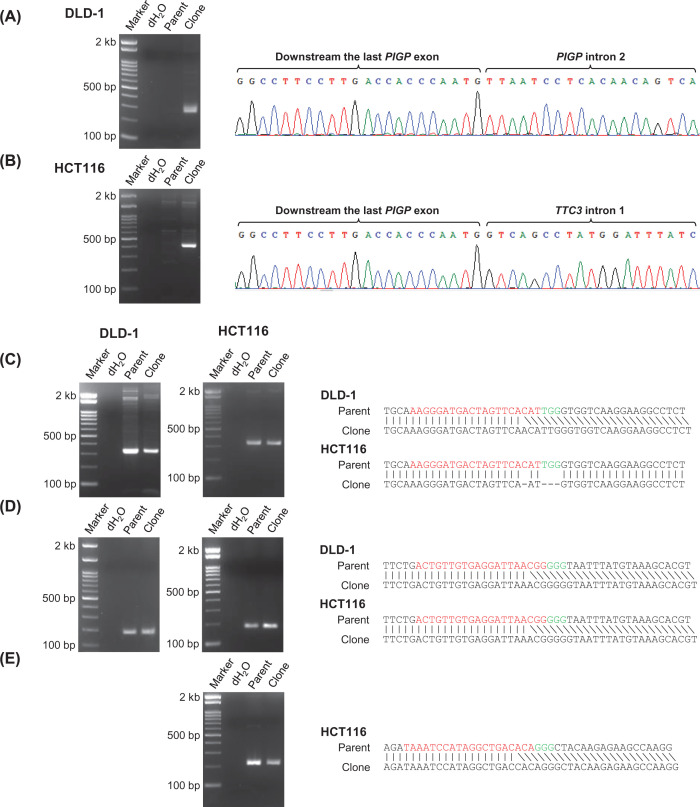
PCRs and DNA sequencing verifying heterozygous deletions in the reporter clones (**A** and **B**) PCR amplifications encompassing the junctions of deletion ends. Shown are PCR products fractionated on agarose gels (left) and their sequencing chromatographs (right) in the heterozygous clones derived from DLD-1 (262 bp; A) and HCT116 (398 bp; B). (**C**–**E**) PCR amplifications encompassing small indels created at Cas9 nuclease target sites. Shown are PCR products on agarose gels (left) and their sequence information (right) at a region downstream from the last *PIGP* exon (C), within *PIGP* intron 2 (D), and within *TTC3* intron 1 (E). PCR products are 320 bp (C), 179 bp (D), and 210 bp (E) in size when amplified from wild-type alleles. Clone: *PIGP*-heterozygous clones; Parent: parental cell controls for *PIGP*-heterozygous clones.

**Figure 4:**
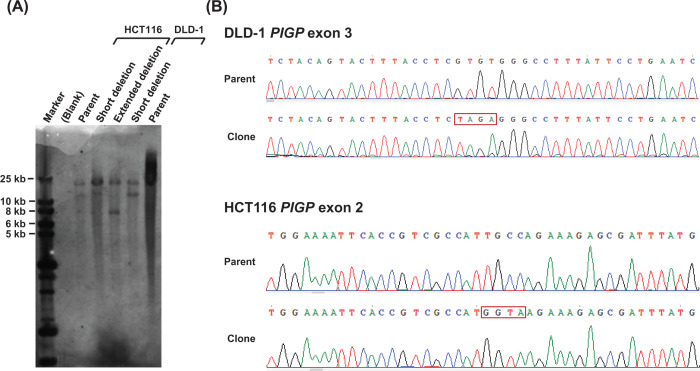
Further validation of the *PIGP*-engineered cell clones (**A**) Southern blot analysis of the *PIGP*-heterozygous clones. The *PIGP* alleles harboring a short (DLD-1 and HCT116) and an extended (HCT116) deletion in *PIGP*-heterozygous clones are supposed to exhibit 12.2- and 7.7-kb bands, respectively, while the wild-type *PIGP* allele should exhibit an 18.4-kb band. For the southern blot experiment, also see Figure 1. (**B**) Sequencing of the genomic regions surrounding the knock-in sites in *PIGP* reporter clones, shown together with those in parental cell controls. Truncating mutations introduced to the *PIGP* reporter clones are marked by red frames on the sequence reads.

To introduce a large deletion within a *PIGP* allele, we initially transfected DLD-1 cells with two plasmids each expressing Cas9 and a sgRNA (a Cas9 nuclease, collectively), which create double-strand breaks (DSBs) within *PIGP* intron 2 and downstream from the last *PIGP* exon, respectively (Figure 2). Puromycin selection was then performed for 48 h to remove untransfected cells. At 14 days after transfection, the selected cells were stained with FLAER and subjected to the FCM-based sorting of FLAER-positive cells to remove cells bearing both *PIGP* alleles disrupted by Cas9 nucleases. Single-cell clones were next isolated from the sorted cell population and processed for PCR screening using three sets of primer pairs; the three PCRs amplified the joint portion of both deletion ends, a genomic region encompassing the Cas9 target site within *PIGP* intron 2, and that downstream from the last *PIGP* exon, respectively (Figures 2 and 3A,C,D). Cell clones indicating positivity in all three PCRs were identified, and PCR products derived from these clones were sequenced. The obtained reads verified the correct joining of deletion ends and the creation of small indels at the Cas9 nuclease target sites that supposedly occurred in the remaining *PIGP* allele. We also performed southern blot analysis to confirm the introduction of the programmed *PIGP*-heterozygous deletion in a representative clone (Figure 4A). This clone is hereafter referred to as a DLD-1-derived *PIGP*-heterozygous clone.

Next, we sought to create a reporter clone that allows us to monitor the correction of a *PIGP*-inactivating mutation by introducing a truncating mutation into the remaining *PIGP* allele in the DLD-1-derived *PIGP*-heterozygous clone. To this end, we transfected the *PIGP*-heterozygous clone with two plasmids each expressing Cas9 (D10A) and a sgRNA (a Cas9 nickase, collectively), together with a donor plasmid carrying the *PIGP* genomic sequence harboring a truncating mutation at exon 3 (Figure 2). This transfection should elicit targeted knock-in of the *PIGP* truncating mutation into the *PIGP*-heterozygous cell clone via TPN, a nick-based strategy for precision targeted knock-in [13]. We thus stained the transfected cells with FLAER and collected FLAER-negative cells by FCM-based cell sorting to obtain a cell population in which both *PIGP* alleles were inactivated by a deletion and a mutation, respectively. The sorted cells were subjected to single-cell cloning, and the knock-in site at *PIGP* exon 3 in the single-cell clones was sequenced (Figure 4B). These procedures led to the identification of cell clones in which the programmed mutation had been incorporated into the intended genomic position. The absence of functional *PIGP* alleles in the identified clones was confirmed by cell staining with FLAER followed by FCM analysis. The cell clones were thereby established as DLD-1-derived *PIGP* reporter clones, which were to be employed for detecting targeted knock-in events.

### Creation of a HCT116-derived reporter clone with which to monitor the correction of a *PIGP* mutation

To reduce biases in measuring the efficiency of targeted knock-in caused by potential cell line-specific and genomic site-specific factors, we sought to engineer another human cell line and create a second series of *PIGP* reporter clones bearing a mutation at a different portion of the *PIGP* gene. Given that a mutation on exon 3 was created in the DLD-1-derived *PIGP* reporter clone, we planned to introduce a distinct truncating mutation on exon 2 into another near-diploid human cell line, HCT116 (Figure 1).

We initially performed the same experimental procedures as described above with the HCT116 cell line to introduce the identical heterozygous *PIGP* deletion with the DLD-1-derived reporter clone (Figure 2). The resulting HCT116-derived clone was next transfected with two Cas9 nucleases targeted to the junction of deletion ends and intron 1 of the *TTC3* gene, respectively. *TTC3* is a gene located next to *PIGP* with a head-to-head configuration, and exon 1s of both genes are located very close to each other (approximately 100 bp apart). If a Cas9 nuclease was designed at a genomic site between two exon 1s, the promoter of the remaining *PIGP* allele would likely be disrupted by indels created at the Cas9-cleaved site, which would compromise the functionality of *PIGP* reporter clones generated through subsequent engineering steps. We thus chose to place one of the Cas9 nucleases within *TTC3* intron 1. This process would extend the original heterozygous deletion in the HCT116-derived clone; now, the deletion should cover the entire *PIGP* gene in addition to *TTC3* exon 1 (Figures 1 and 2). After transfection with the two Cas9 nucleases, cells were selected with puromycin for 48 h, stained with FLAER at 10 days after transfection, and subjected to FCM-based sorting to collect FLAER-positive cells. Single-cell clones were isolated from the collected cells and then analyzed for their *PIGP* gene statuses by four different analytical PCRs: one encompassing the extended deletion and the others encompassing the Cas9 nuclease target sites located within *TTC3* intron 1, within *PIGP* intron 2, and downstream from the last *PIGP* exon, respectively (Figures 2 and 3B–E). Cell clones positive for all four analytical PCRs were identified, and the existence of a functional *PIGP* gene in these clones was confirmed by cell staining with FLAER followed by FCM analysis. Southern blot analysis was performed to confirm the genomic structure at the *PIGP* locus in one of the clones (Figure 4A). This clone is hereafter referred to as a HCT116-derived *PIGP*-heterozygous clone.

We next employed the HCT116-derived *PIGP*-heterozygous clone to create a reporter clone allowing us to monitor the efficiency of targeted knock-in by introducing a truncating mutation into exon 2 within the remaining *PIGP* allele. We chose to accomplish this by TPN-based targeted knock-in and transfected the HCT116-derived *PIGP*-heterozygous clone with two Cas9 nickases targeted to the region surrounding the knock-in site, along with a donor plasmid carrying a *PIGP* sequence harboring a truncating mutation on exon 2 (Figure 2). The cells were then stained with FLAER and subjected to FCM-based sorting to collect the FLAER-negative cells. Single-cell clones were isolated from the collected cells and processed for Sanger sequencing to verify the targeted incorporation of the programmed *PIGP* mutation in these clones (Figure 4B). After the confirmation of FLAER-negativity by FCM analysis, these cell clones were established as HCT116-derived *PIGP* reporter clones for monitoring targeted knock-in events.

### Measuring the efficiency of targeted knock-in using the *PIGP* reporter clones

**Figure 5:**
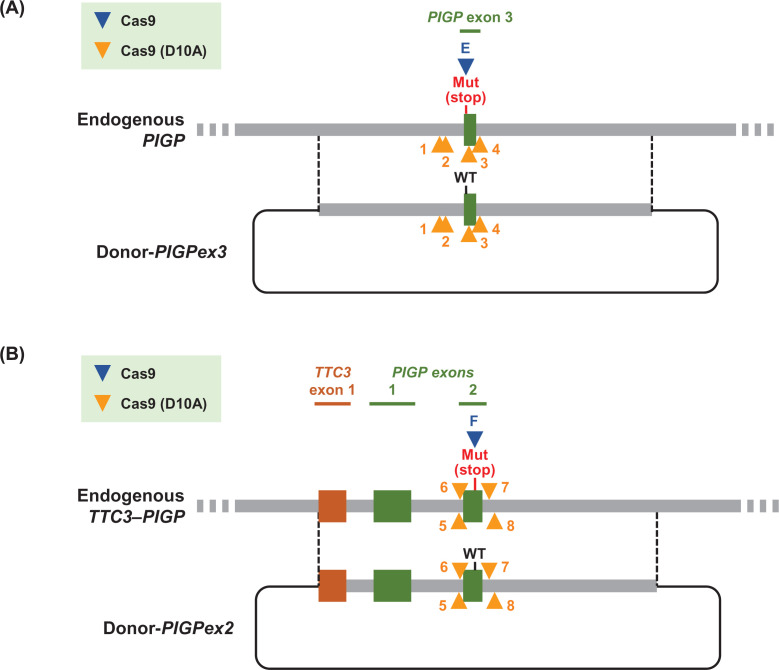
Schemes depicting the targeted correction of *PIGP* truncating mutations Truncating mutations within *PIGP* exon 3 (DLD-1) (**A**) and exon 2 (HCT116) (**B**) were corrected. Tandem Cas9 nickase pairs were transfected into cells together with a donor plasmid to perform TPN-based targeted knock-in. To achieve conventional DSB-mediated targeted knock-in, a Cas9 nuclease and a donor plasmid were transfected into cells. Dotted lines indicate regions of homology between the genome and donor plasmids. Mut (stop): truncating mutation.

**Figure 6:**
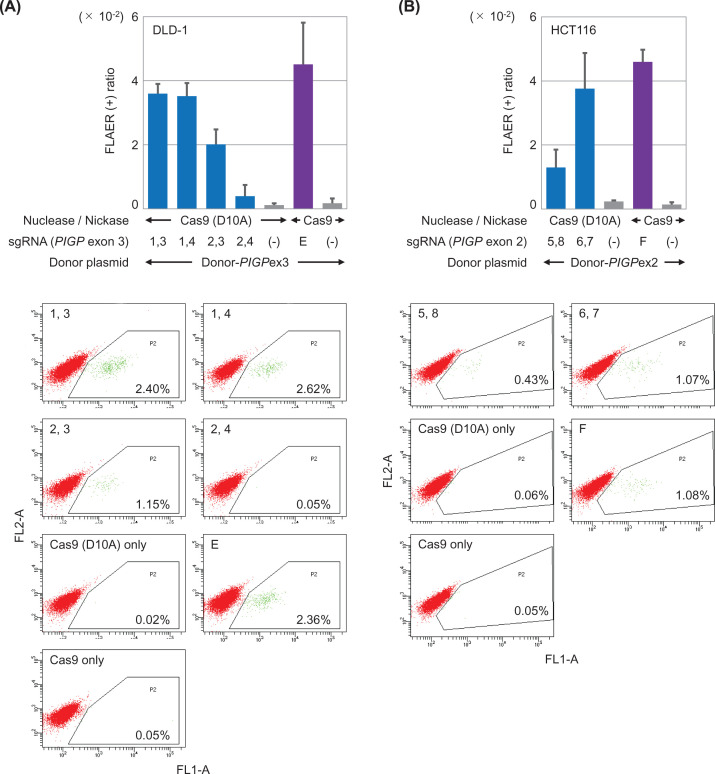
Efficiency of *PIGP* gene correction achieved via the TPN strategy and the Cas9 nuclease-based strategy Shown are mean and SD values of data obtained in three independent experiments (top) and representative dot graphs (bottom) in DLD-1-derived (**A**) and HCT116-derived (**B**) *PIGP* reporter clones. Percentages of FLAER-positive cells in respective samples are noted in dot graphs.

We next compared the efficiency of targeted knock-in achieved via the TPN strategy versus a conventional Cas9 nuclease-based strategy using the *PIGP* reporter clones as platforms of the assay. For this purpose, we initially transfected the DLD-1-derived and HCT116-derived *PIGP* reporter clones with donor plasmids carrying the relevant portions of the wild-type *PIGP* sequence (Donor-*PIGP*ex3 and Donor-*PIGP*ex2, respectively) together with tandem paired Cas9 nickases or a Cas9 nuclease (Figure 5). Cells were then stained with FLAER and analyzed by FCM. These assays, namely *PIGP* correction assays, demonstrated that half of the Cas9 nickase pairs used in the TPN strategy served to achieve efficiencies of targeted knock-in largely comparable to that achieved by Cas9 nucleases (Figure 6). These data are consistent with our previous findings obtained via *PIGA* correction assays [13], suggesting that the *PIGP* correction assay is useful as a second assay measuring the efficiency of targeted knock-in similar to the *PIGA* correction assay.

### The use of dual sgRNA vectors enhances the efficiency of targeted knock-in via TPN

**Figure 7:**
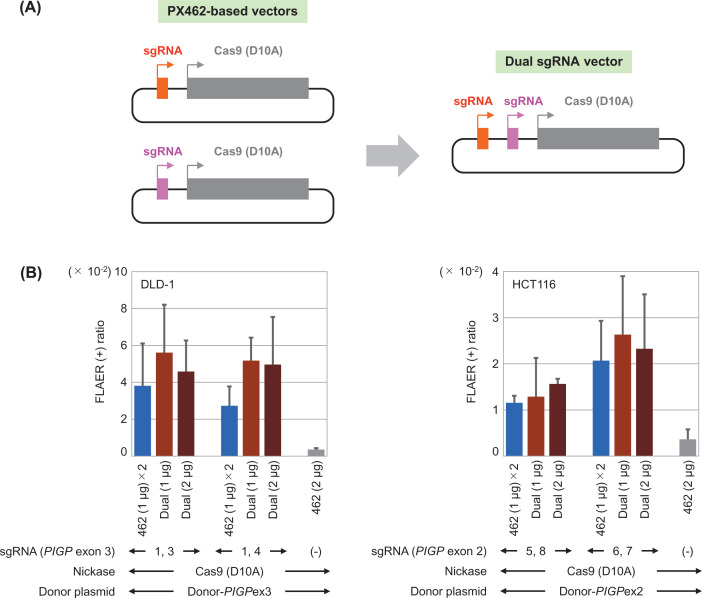
The use of dual sgRNA vectors marginally enhances targeted knock-in via TPN (**A**) Construction of a dual sgRNA vector. (**B**) Efficiencies of targeted knock-in via TPN using PX462-based vectors (blue) and those using dual sgRNA vectors (light and dark brown). Mean and SD values of three independent experiments are shown in the graphs. The use of 1 μg dual sgRNA vectors (light brown bars) significantly increased the efficiency of targeted knock-in compared with the use of PX462-based vectors (blue bars) only in the DLD-1-derived reporter clone (*P*=0.001). Neither of the reporter clones indicated significant differences in the achieved efficiency of targeted knock-in between the use of PX462-based vectors (blue bars) and 2 μg dual sgRNA vectors (dark brown bars), and the use of 1 μg and 2 μg dual sgRNA vectors (light and dark brown bars, respectively). 462: PX462-based vector; dual: dual sgRNA vector.

We lastly sought to harness the *PIGP* correction assay to improve the targeted knock-in procedure. We and others have demonstrated the utility of TPN to conduct precise and relatively efficient targeted knock-in [13,26,27]. Although two sgRNAs used for TPN in these studies were transfected into cells using two plasmids each expressing Cas9 (D10A) and a sgRNA, we postulated that the use of all-in-one vectors expressing two sgRNAs and Cas9 (D10A) from a single plasmid would allow the efficient simultaneous expression of all these genome editing tools within recipient cells and would thereby help achieve higher efficiency of targeted knock-in via TPN. We therefore created plasmids harboring three gene cassettes expressing a Cas9 (D10A) and two sgRNAs targeted near the *PIGP* knock-in site, respectively (Figure 7A). Similar all-in-one vectors expressing up to seven sgRNAs and a Cas9 nuclease were previously created and employed for multiplex DSB-mediated genome engineering in human cells [28]. The plasmids that we created, named dual sgRNA vectors, were transfected into DLD-1-derived and HCT116-derived reporter clones; the transfected clones were then subjected to *PIGP* correction assays. As a result, the average efficiencies of the targeted knock-in achieved using dual sgRNA vectors were 1.1- to 1.9-fold higher than those obtained with two PX462-based expression vectors in all comparisons performed using a total of four tandem Cas9 nickase pairs with DLD-1-derived and HCT116-derived reporter clones (Figure 7B). However, the differences reached statistical significance only in half of the dataset derived from the DLD-1-derived reporter clone. Overall, the data obtained in these assays demonstrated that the use of a dual sgRNA vector slightly enhances the efficiency of targeted knock-in achieved via TPN compared with the use of two PX462-based expression vectors.

## Discussion

In the present study, we created reporter clones in which two *PIGP* alleles were inactivated by a genomic deletion and a truncating mutation, respectively. The reporter clones were employed to perform the *PIGP* correction assay that monitors the correction of *PIGP* mutations achieved via targeted knock-in. Although a previously established *PIGA* reporter clone used in the *PIGA* correction assay derives from a single cell line (HCT116) [13], the *PIGP* reporter clones derive from two cell lines (DLD-1 and HCT116), and these clones have truncating mutations on different *PIGP* exons. Therefore, the use of the *PIGP* correction assay will help reduce concerns for genomic site-specific and cell line-specific biases that potentially affect the outcomes of the assays. In addition, the joint use of *PIGA*- and *PIGP*-based assays will further provide variety in genomic loci employed as assay platforms.

Similar to the *PIGA* correction assay, the *PIGP* correction assay monitors the efficiency of targeted knock-in harnessing a GPI-anchor biosynthesis gene as a reporter. In both of these assays, GPI anchors protruding from the cell membrane are detected as a surrogate marker for the presence of a functional reporter gene. FCM-based quantification of GPI-anchor-positive cells has been well established as a sensitive, simple, and relatively inexpensive methodology to determine the efficiency of targeted knock-in in the *PIGA* correction assay, and the *PIGP* correction assay shares the same benefits.

We initially created large heterozygous deletions within and over the *PIGP* gene to create the *PIGP* reporter clones. This initial deletion step enables the use of autosomal genes, such as the *PIGP* gene, to quantify the efficiency of targeted knock-in. Other GPI-anchor biosynthesis genes, including *PIGL* [17], will also be useful as platform genes for FCM-based detection of targeted knock-in events, once a heterozygous deletion is initially created. In addition, although the cell lines employed in the present study (DLD-1 and HCT116) are of male origin, it should also be possible to use female cell lines for an assay monitoring gene correction events by first introducing the heterozygous deletion of a reporter gene. Thus, the initial deletion step will be able to further increase the choice of reporter genes and platform cell lines in monitoring the efficiency of targeted knock-in. In summary, our current study demonstrated that the *PIGP* correction assay is useful for measuring the efficiency of targeted knock-in and, together with the *PIGA* correction assay, will help further improve the efficiency of CRISPR/Cas-assisted targeted knock-in.

## Conclusion

Collectively, the present study has served to extend the variety of reporter genes and platform cell lines harnessed to measure the efficiency of targeted knock-in and will thereby help minimize the biases in the observed efficiency of targeted knock-in caused by locus-specific and cell line-specific factors.

## Supplementary Material

Supplementary Figures S1-S2 and Tables S1-S2

## Data Availability

Data and materials will be made available from the corresponding author upon reasonable request.

## Abbreviations

DSB: double-strand break
FBS: fetal bovine serum
FCM: flow cytometry
FLAER: fluorescent-labeled inactive toxin aerolysin
gDNA: genomic DNA
GPI: glycosylphosphatidylinositol
P&S: penicillin and streptomycin
PAM: protospacer adjacent motif
PCR: polymerase chain reaction
sgRNA: single guide RNA
TPN: tandem paired nicking
