# Supplementary material for: Flow cytometry-based quantification of targeted knock-in events in human cell lines using a GPI-anchor biosynthesis gene PIGP
Source: Biosci Rep. 2021 Dec 8;41(12):BSR20212231. doi: 10.1042/BSR20212231 (PMC8661509; doi:10.1042/BSR20212231)
Supplement: Supplementary Figures S1-S2 and Tables S1-S2 [file BSR-2021-2231_supp.pdf]

Supplementary Information

for

Flow cytometry-based quantification of targeted knock-in events in human cell lines using a GPI-anchor biosynthesis gene *PIGP*

Md. Lutfur Rahman, Toshinori Hyodo, Muhammad Nazmul Hasan, Yuko Mihara, Sivasundaram Karnan, Akinobu Ota, Shinobu Tsuzuki, Yoshitaka Hosokawa, and Hiroyuki Konishi*

Department of Biochemistry, Aichi Medical University School of Medicine, Nagakute, Aichi 480-1195, Japan

* Corresponding author
Hiroyuki Konishi
Department of Biochemistry, Aichi Medical University School of Medicine
1-1 Yazako Karimata, Building #2, Room 362,
Nagakute, Aichi 480-1195, Japan
Phone: +81-561-62-3311 ext. 12362
Fax: +81-561-61-4056
E-mail: hkonishi@aichi-med-u.ac.jp

- **Supplementary Figures 1 and 2**
- **Supplementary Tables 1 and 2**

Supplementary Figure 1

Donor-*PIGP*ex3

```
−174        #1      −154                #2      −127                                                 −75
--aaacgagcta gaagaactga aaggaggaaa aaatcatttc tgagttaatc agggacactt caaaagagct tttctgtttt atctgaatct taatagattg
--tttgctcgat cttcttgact ttcctccttt tttagtaaag actcaattag tccctgtgaa gttttctcga aaagacaaaa tagacttaga attatctaac

 −74                                                                           ±0          #3        +19  +25
  atagattaga tgtatgtctt actgacttct cttcctcttt tttttctttt tcttttctac agTACTTTAC CTCGTGTGGG CCTTTATTCC TGAATCTTGG
  tatctaatct acatacagaa tgactgaaga gaaggagaaa aaaaagaaaa agaaaagatg tcATGAAATG GAGCACACCC GGAAATAAGG ACTTAGAACC
                                                                        I  L  Y   L  V  W   A  F  I  P   E  S  W

 +26                                                  #4        +82                                        +125
  CTAAACTCTT TAGGTTTAAC CTATTGGCCT CAAAAgtaag tttaatatat ttcttaaatg aggatataat taatgctgtt ttgaggagtc atatcatttc--
  GATTTGAGAA ATCCAAATTG GATAACCGGA GTTTTcattc aaattatata aagaatttac tcctatatta attacgacaa aactcctcag tatagtaaag--
   L  N  S   L  G  L  T   Y  W  P   Q  K
```

Genomic region surrounding *PIGP* exon 3

```
−174        #1      −154                #2      −127                                                 −75
--aaacgagcta gaagaactga aaggaggaaa aaatcatttc tgagttaatc agggacactt caaaagagct tttctgtttt atctgaatct taatagattg
--tttgctcgat cttcttgact ttcctccttt tttagtaaag actcaattag tccctgtgaa gttttctcga aaagacaaaa tagacttaga attatctaac

                                                                           E      −1 ±0
 −74                                                                                       #3        +19  +25
  atagattaga tgtatgtctt actgacttct cttcctcttt tttttctttt tcttttctac agTACTTTAC CTCTAGAGGG CCTTTATTCC TGAATCTTGG
  tatctaatct acatacagaa tgactgaaga gaaggagaaa aaaaagaaaa agaaaagatg tcATGAAATG GAGATCTCCC GGAAATAAGG ACTTAGAACC
                                                                        I  L  Y   L  *  R   A  F  I  P   E  S  W

 +26                                                  #4        +82                                        +125
  CTAAACTCTT TAGGTTTAAC CTATTGGCCT CAAAAgtaag tttaatatat ttcttaaatg aggatataat taatgctgtt ttgaggagtc atatcatttc--
  GATTTGAGAA ATCCAAATTG GATAACCGGA GTTTTcattc aaattatata aagaatttac tcctatatta attacgacaa aactcctcag tatagtaaag--
   L  N  S   L  G  L  T   Y  W  P   Q  K
```

Supplementary Figure 1 DNA sequences surrounding the knock-in site at *PIGP* exon 3 in the Donor-*PIGP*ex3 plasmid (top) and the DLD-1-derived *PIGP* reporter clone (bottom). Target sequences of a Cas9 nuclease and Cas9 nickases are shaded in blue and green, respectively, with darker shading on their neighboring protospacer adjacent motifs (PAMs). Numbers with plus or minus signs near Cas9 target sites indicate the distance from the knock-in site (±0) to theoretical cleavage sites within individual Cas9 target sites. Nucleotides shown by red and green bold letters represent a *PIGP* truncating mutation in the DLD-1-derived *PIGP* reporter clone and its corresponding wild-type sequence in Donor-*PIGP*ex3, respectively. Upper case and lower case letters indicate exonic and intronic sequences, respectively.

Supplementary Figure 2

Donor-*PIGPex2*

```
−134                                    #5         −93                                                          −35
--gtacccgcgg cggagcgagg aggcgagaat ggatcaatgg tgccacggag cacatcgctg acgctgattg tgttcctttt ccacagATTG TCTAAAGCCC
--catgggcgcc gcctcgctcc tccgctctta cctagttacc acggtgcctc gtgtagcgac tgcgactaac acaaggaaaa ggtgtcTAAC AGATTTCGGG
                                                     −86        #6

−34                                        ±0                                                                  +65
  CAGGAAAAAT GGTGGAAAAT TCACCGTCGC CATTGCCAGA AAGAGCGATT TATGGCTTTG TTCTTTTCTT AAGCTCCCAA TTTGGCTTCA gtaagtactt
  GTCCTTTTTA CCACCTTTTA AGTGGCAGCG GTAACGGTCT TTCTCGCTAA ATACCGAAAC AAGAAAAGAA TTCGAGGGTT AAACCGAAGT cattcatgaa
          M   V  E  N   S  P  S   P  L  P  E   R  A  I   Y  G  F   V  L  F  L   S  S  Q   F  G  F

+66                                                #8        +118                                              +165
  tcctgatgga tggccagggg ctttttctgg cgttcagtga caagggaaga atacattggt tagactgtgc acacatgaac tgttgattct ttctccctat--
  aggactacct accggtcccc gaaaaagacc gcaagtcact gttcccttct tatgtaacca atctgacacg tgtgtacttg acaactaaga aagagggata--
                   +85       #7
```

Genomic region surrounding *PIGP* exon 2

```
−134                                    #5         −93                                                          −35
--gtacccgcgg cggagcgagg aggcgagaat ggatcaatgg tgccacggag cacatcgctg acgctgattg tgttcctttt ccacagATTG TCTAAAGCCC
--catgggcgcc gcctcgctcc tccgctctta cctagttacc acggtgcctc gtgtagcgac tgcgactaac acaaggaaaa ggtgtcTAAC AGATTTCGGG
                                                     −86        #6

−34                        F        −6     ±0                                                                  +65
  CAGGAAAAAT GGTGGAAAAT TCACCGTCGC CATGGTAAGA AAGAGCGATT TATGGCTTTG TTCTTTTCTT AAGCTCCCAA TTTGGCTTCA gtaagtactt
  GTCCTTTTTA CCACCTTTTA AGTGGCAGCG GTACCATTCT TTCTCGCTAA ATACCGAAAC AAGAAAAGAA TTCGAGGGTT AAACCGAAGT cattcatgaa
          M   V  E  N   S  P  S   P  W  *  E   R  A  I   Y  G  F   V  L  F  L   S  S  Q   F  G  F

+66                                                #8        +118                                              +165
  tcctgatgga tggccagggg ctttttctgg cgttcagtga caagggaaga atacattggt tagactgtgc acacatgaac tgttgattct ttctccctat--
  aggactacct accggtcccc gaaaaagacc gcaagtcact gttcccttct tatgtaacca atctgacacg tgtgtacttg acaactaaga aagagggata--
                   +85       #7
```

Supplementary Figure 2 DNA sequences surrounding the knock-in site at *PIGP* exon 2 in the Donor-*PIGP*ex2 plasmid (top) and the HCT116-derived *PIGP* reporter clone (bottom). For annotations of the numbers and colors shown in this figure, see the legend for Supplementary Figure 1.

Supplementary Table 1: Cas9 target sites with PAMs

Target name	Sequence
Creation of the initial deletion	
PIGP-nuclease-A	ACTGTTGTGAGGATTAACGGGGG
PIGP-nuclease-B	AAGGGATGACTAGTTCACATTGG
Extension of the deletion in HCT116	
TTC3-nuclease-C	TAAATCCATAGGCTGACACAGGG
Junction-nuclease-D	CTGTTGTGAGGATTAACATTGGG
PIGP correction assay using the DLD-1-derived PIGP reporter clone	
PIGP-nickase-1	GAGCTAGAAGAACTGAAAGGAGG
PIGP-nickase-2	AATCATTTCTGAGTTAATCAGGG
PIGP-nickase-3	GGGCCTTTATTCCTGAATCTTGG
PIGP-nickase-4	TTTAATATATTTCTTAAATGAGG
PIGP-nuclease-E	TACAGTACTTTACCTCTAGAGGG
PIGP correction assay using the HCT116-derived PIGP reporter clone	
PIGP-nickase-5	AGAATGGATCAATGGTGCCACGG
PIGP-nickase-6	AGCGTCAGCGATGTGCTCCGTGG
PIGP-nickase-7	TGAACGCCAGAAAAAGCCCCTGG
PIGP-nickase-8	GTGACAAGGGAAGAATACATTGG
PIGP-nuclease-F	TGGAAAATTCACCGTCGCCATGG

PAM, protospacer adjacent motif

Underlining indicates PAM sequences.

Supplementary Table 2: PCR primers generated in this study

Primer name	Sequence
Creation of dual sgRNA vectors	
U6 promoter-F	5′-CCTAGGTCTAGAGAGGGCCTATTTCCCATGATTCC
CAG-R	5′-CCATTTACCGTAAGTTATGTAAC
Creation of Donor-PIGPex3	
*PIGP*int2-F2	5′-CTTTTAGAGCTCATTCGTGAGCACTTGTAA
*PIGP*int3-R	5′-TTTTCTGGATCCTGCATTATCTCTATTTCCTT
Creation of Donor-PIGPex2	
*TTC3*ex1-R	5′-CTCGGCTGCAGCGGGACAA
*PIGP*int2-R1	5′-CCTACCCCATAGAACTGTTTCATGCGTC
Analytical PCRs probing deletions	
*PIGP*int2-F1	5′-TGGCTGACTTCACTCTGCTG
*PIGP*int2-R2	5′-AGCTTCTAATCCTTGACATACTCTT
*PIGP*downstream-F1	5′-CAGGGTGAGGCAACTAAGCA
*PIGP*downstream-R1	5′-CCAGCCTACAAAGCCCTTCA
*TTC3*int1-F	5′-GCCACCTTGGCTTCTCTTGT
*TTC3*int1-R	5′-CACCTCACCTGCCACTAAAG
Production of PIGP Southern probe	
*PIGP*downstream-F2	5′-CTGAGTTTTGGTCAAGCAGGG
*PIGP*downstream-R2	5′-ACACTAAACTGATCATGACCCTCA

PCR, polymerase chain reaction

Underlining indicates the restriction enzyme sites used for the incorporation of PCR products into a plasmid.
